# The First Mutation Identified in a Chinese Acrodysostosis Patient Confirms a p.G289E Variation of *PRKAR1A* Causes Acrodysostosis

**DOI:** 10.3390/ijms150813267

**Published:** 2014-07-29

**Authors:** Nan Li, Min Nie, Mei Li, Yan Jiang, Xiaoping Xing, Ou Wang, Chunlin Li, Weibo Xia

**Affiliations:** 1 Department of Endocrinology, Key Laboratory of Endocrinology, Ministry of Health, Peking Union Medical College Hospital, Chinese Academy of Medical Science, Beijing 100730, China; E-Mails: wsln_may07@163.com (N.L.); niem@pumch.cn (M.N.); limeilzh@sina.com (M.L.); sinojenny@126.com (Y.J.); xingxp2006@126.com (X.X.); wangou1973@sohu.com (O.W.); 2 Department of Geriatric Endocrinology, Chinese PLA General Hospital, Beijing 100853, China; E-Mail: lcl301@sina.cn

**Keywords:** rare skeletal dysplasia, Chinese, the cyclic AMP (cAMP) signaling, hormone resistance, PRKAR1A

## Abstract

Acrodysostosis is a rare skeletal dysplasia, which has not been reported previously in patients of Chinese origin. The *PRKAR1A* gene and *PDE4D* gene have been found to be causative genes of acrodysostosis. A Chinese girl with acrodysostosis and concomitant multiple hormone resistance was recruited for our study. Clinical and biochemical characters were analyzed. DNA was extracted from leukocytes and was sequenced for *GNAS*,* PDE4D* and* PRKAR1A* gene mutations. A *de novo* heterozygous missense mutation (c.866G>A/p.G289E) was identified in the *PRKAR1A* gene. This mutation coincided with a mutation that had been found in a patient from another ethnic group. Our findings further suggest that the c.866G>A/p.G289E mutation in the *PRKAR1A* gene may be the cause of acrodysostosis with concomitant multiple hormone resistance. Moreover, it is the first report of acrodysostosis genetic analysis of Chinese origin.

## 1. Introduction

Acrodysostosis (online Mendelian inheritance in man number (MIM) 101800) is a rare syndrome that is characterized by severe brachydactyly, facial dysostosis, nasal hypoplasia, and short stature. These patients always have advanced skeletal maturation and are often overweight. Because their skeletal abnormalities resemble those observed with Albright hereditary osteodystrophy (AHO, MIM number 103580), a final diagnosis must be made based on fine clinical differentiation and sometimes by excluding mutations in the *GNAS* gene.

The first cases of acrodysostosis were described by Maroteaux and Malamut in 1968 [1], and it was defined as a new clinical and radiological entity by Robinow *et al*., in 1971 [2]. Acrodysostosis is believed to be an autosomal dominant condition [3,4]. Resistance to multiple hormones, including parathyroid hormone and thyrotropin, has been reported in some patients with acrodysostosis, but not in all of these patients.

Mutations in the *PRKAR1A* (MIM 188830) gene have been identified in several cases of acrodysostosis with concomitant resistance to multiple hormones [5,6]. The *PRKAR1A* gene is located on chromosome 17q23-q24 and comprises 12 exons that span approximately 20 kb. The coding region, which is 1146 bp in size, encompasses 3 to 12 exons and yields a 381-amino acid protein that is referred to as PRKAR1A. PRKAR1A (cAMP-dependent protein kinase type I-alpha regulatory subunit) is the type I-alpha regulatory subunit (RIα) of cAMP-dependent protein kinase (PKA) and is important for the PKA system to self-regulate its assembly, respond to cAMP, and its disassembly [7]. Accordingly, mutations affecting *PDE4D* encoding phosphodiesterase (PDE) 4D, a class IV cAMP-specific PDE that hydrolyzes cAMP, were recently reported in a subset of acrodysostostic patients [6].

Here, we report on a de novo *PRKARA1* mutation in a Chinese girl with acrodysostosis and hormone resistance.

## 2. Results and Discussion

### 2.1. Results

A 12-year-old girl was referred to our clinic because of brachydactyly. She had been a full-term infant and had been delivered after an uncomplicated pregnancy. At birth, she weighed 2600 g and was 46 cm in length. Her neonatal period was uncomplicated. Her parents did not show any visible signs of AHO. She was an average kid in her class, indicating that she had normal intelligence.

**Figure 1:**
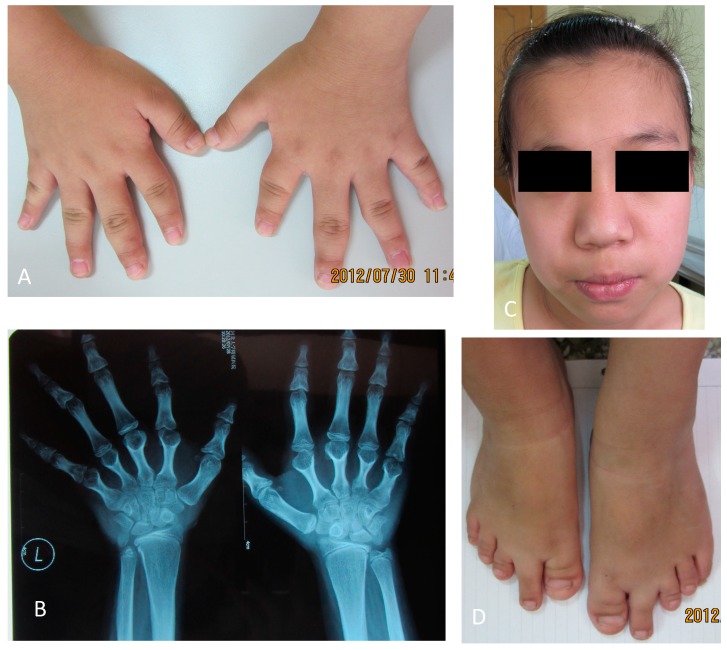
Patient photographs and X-ray images. (**A**) Small hands; (**B**) X-ray of the patient’s hands shows shortened metacarpals; (**C**) Round face and “pug-nose” can be observed in the patient’s photograph; and (**D**) Photograph of the patient’s feet shows shortened toes and metatarsals.

On physical examination, she was 141 cm high (−2 SD for a normal Chinese girl; Median length of 12-year-old Chinese girl is 152.4 cm, with SD of about 6.5 cm) and weighed 39 kg (at the average level of normal Chinese school children; the median weight of a 12-year-old Chinese girl is 40.77 kg). Her father was 182 cm tall and her mother was 168 cm tall. She displayed typical somatic and skeletal features of Albright’s hereditary osteodystrophy with a round face and shortened metacarpals and metatarsals. We also observed maxillofacial hypoplasia with flattening of the nasal ridge associated with small and upturned nostrils (Figure 1). Subcutaneous calcifications were not observed.

**Figure 2:**
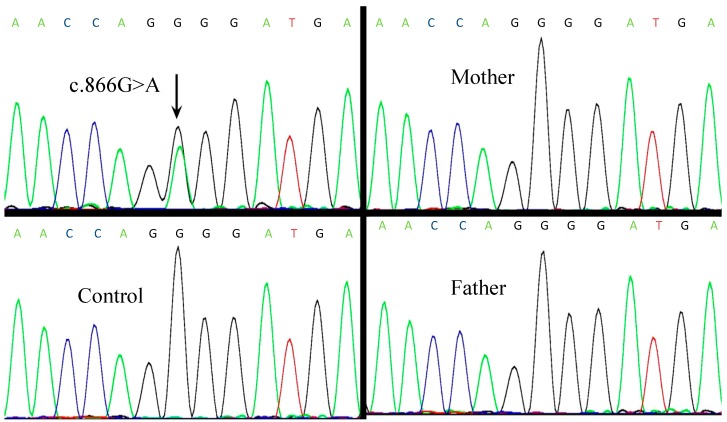
Automated sequencing traces for *PRKAR1A* gene mutations. (**A)** Sequencing trace for the patient shows a heterozygous c.866G>A mutation; (**B**–**D**) Control and the patient’s parents sequencing traces show no abnormalities at the same site.

Skeletal maturation assessment by plain radiographs revealed shortened metacarpals and metatarsals. Her bone age was estimated to be 13 years. All biochemical measurements (results in Table 1) were performed using standard clinical laboratory procedures for electrolyte and hormone concentrations in blood and urine. Her serum calcium (Ca), phosphate (Pi), and alkaline phosphatase (ALP) were within normal ranges. Urinary calcium excretion (uCa) was normal. Her serum parathyroid hormone (PTH) was high, which indicated PTH resistance. She had normal free triiodothyronine (FT3) and free thyroxin (FT4) levels in association with increased thyrotropin (TSH) levels, which indicated TSH resistance. In the mutation analysis, we found a missense mutation in exon 8 (c.866G>A), which had generated a substitution of the amino acid glycine (GGG) to glutamic acid (GAG) at codon 289 (p.G289E). This mutation was not found in the patient’s parents, which suggested that this mutation had occurred *de novo* in this patient. DNA sequence abnormalities, which could be identified by comparisons with the reference sequence (RefSeq accession number: NM_002734.3), were absent in the 100 unrelated healthy controls (50 males and 50 females), which indicated that this variation was not a single nucleotide polymorphism (SNP) (Figure 2).

**Table 1:** Biochemical measurements of the patient.

Items	Patient’s Value	Reference Range
Serum Ca (mmol/L)	2.33	2.13–2.7
Serum Pi (mmol/L)	1.81	1.13–1.87 (for children)
ALP (U/L)	219	42–390
PTH (pg/mL)	**107**	12–65
FT3 (pg/mL)	3.91	1.8–4.1
FT4 (ng/dL)	1.18	0.81–1.89
TSH (μIU/mL)	**7.902**	0.38–4.34
IGF-1 (ng/mL)	576	111–551
24 h-uCa (mmol)	0.76	-
24 h-uP (mmol)	29.22	-

Abnormal numbers showed in bold characters. ALP, alkaline phosphatase; PTH, parathyroid hormone; FT, free triiodothyronine; TSH, thyrotropin; IGF-1, insulinlike growth factor-1; uCa, urinary calcium excretion; uP, urinary phosphate excretion.

### 2.2. Discussion

Acrodysostosis is an autosomal dominant genetic disease that often occurs sporadically. Our patient had acrodysostosis concomitant with hormone resistance and had no family history of this disorder. We found a heterozygous *de novo* mutation that resulted in the amino acid substitution p.G289E in PRKAR1A.

PKA is the primary mediator of cAMP signaling in mammals and plays a major role in eukaryotic cell signaling. The PKA holoenzyme comprises two catalytic (C) subunits and two regulatory (R) subunits. These form a tetramer, R2C2, which dissociates in the presence of cAMP into an R2(cAMP)4 dimer and two free catalytically active C subunits. To date, four major R subunit isoforms (RIα, RIβ, RIIα, and RIIβ) and three C subunit isoforms (Cα, Cβ, and Cγ) have been identified. PRKAR1A is the RIα subunit of PKA.

It has been confirmed that an R subunit is essential for the PKA system to self-regulate its assembly, respond to cAMP, and its disassembly [7]. Gene knockout (KO) mice have provided insights into the specific functions of PKA with regard to its associations with various R subunit isoforms. RIα-KO mice display the most severe phenotype, which indicates the essential role of the RIα regulatory subunit in maintaining proper PKA activity during physiological processes [8,9]. The RIα subunit exerts its function via maintaining the catalytic subunit under cAMP control and, possibly, via other unknown means outside the PKA holoenzyme.

RIα has a conserved, well-defined domain structure comprising an amino-terminal dimerization/docking domain, two tandem cAMP-binding domains at its carboxyl terminus, and a variable, interconnecting linker region containing the primary docking site for the C-subunit. RIα has a nearly ubiquitous distribution; thus disrupting RIα may result in resistance to various hormones that depend on cAMP signaling [10].

To date, only 22 patients with acrodysostosis have been reported to have *PRKAR1A* mutations. Among them, 13 had the recurrent p.R368X mutation and the other 9 had different mutations [5,6,11,12]. *PRKAR1A* mutations have also been found in patients with primary pigmented nodular adrenocortical disease (PPNAD), Carney Complex (CNC), and sporadic tumors. However, the mutations in these cases were found to induce PKA activity. The definite mechanisms have not yet been determined.

The cAMP binding domains of RIα are the most conserved portions of the cAMP binding domains [7]. The location of the mutation in the present study suggests that it mediates a change in the conformation of the PRKAR1A protein and results in reduced PRKAR1A function for binding to cAMP. This hypothesis was indirectly confirmed by using an online protein prediction program. We hypothesize that our mutation possibly changes the conformation of this protein and subsequently alters proper PKA enzymatic function.

Linglart* et al.* [12] reported the same mutation that we identified. The clinical features of their patient who had the p.G289E mutation were quite similar to those of our patient, including facial dysostosis, severe brachydactyly, and normal intelligence. Our findings further confirmed that the p.G289E variation could be a cause of acrodysostosis. In addition, the p.289glycine is very important for PRKAR1A function. Coincidently, the same position mutation p.G289W was previously identified in a patient with Carney Complex [13]. It was also a heterozygous site mutation (c.865G>T) that changed the function of the PRKAR1A protein.

These patients exhibit increased PKA-specific activation compared to controls (2.92-fold higher). Different from our mutation, the mutation in Carney Complex was a polar, electrically-neutral glycine that was substituted by a nonpolar amino acid, while our mutation was a polar, electrically-neutral glycine that was substituted by a polar acidic amino acid, glutamic acid. Although we did not test PKA activity for our patient, the different kinds of amino acid substitution may have different results in terms of protein conformation and function, as well as the possibility of RIα function outside the PKA holoenzyme.

Acrodysostosis and pseudohypoparathyroidism type 1a (PHP1a) with AHO share some clinical features. However, acrodysostosis patients have more severe skeletal dysplasia. In addition, mental retardation, heterotopic ossifications, and hypocalcaemia are always absent in acrodysostosis. Urinary cAMP always increase sharply in acrodysostosis patients in response to exogenous parathyroid hormone [5], while PHP1a patients have no response. Although we did not test urinary cAMP level in our patient, clinical manifestations can provide some clues for a clinical diagnosis. Ideally, a genetic analysis is crucial for a final diagnosis. PHP1a was excluded from our patient’s diagnosis based on her negative findings for *GNAS* gene mutations.

There were some limitations for our study. First, the cAMP levels and PKA activity of this patient were not tested. Second, although we predicted mutant protein function using an online program, functional experiments may be more convincing. Further investigation on PRKAR1A activity will be required.

## 3. Experimental Section

### 3.1. Mutation Analysis

Informed consent was signed by the patient’s parents prior to our investigation. Venous blood samples were collected from the patient, her parents, and 100 healthy controls. Leukocytes DNA was extracted using QIAGEN DNA mini kits (Qiagen, Hilden, Germany). *GNAS* gene analysis was done first and showed no abnormalities (data not shown).

Then, in the following analysis of *PRKAR1A* gene and *PDE4D* gene, a *PRKAR1A* gene abnormality was detected in this patient by polymerase chain reaction (PCR) using the primers shown in Table S1. The full coding sequence and the exon-intron boundaries of the *PRKAR1A* gene were analyzed. PCR products were gel purified, and DNA sequences were determined by Taq polymerase cycle sequencing with a semi-automated detection system (ABI 373XL Sequencer; Applied Biosystems, Foster City, CA, USA).

A *de novo* heterozygous missense mutation (c.866G>A/p.G289E) in the *PRKAR1A* gene was identified in the patient. The p.289 glycine was highly evolutionarily conserved among various species based on a database analysis at the University of California Santa Cruz (UCSC) Genome Bioinformatics website [14]. (Table 2).

**Table 2:** p.289 glycine highly conserved among various species. G in red shows highly conserved p.289 glycine from fish to humans.

Species	Amino Acid Sequence							
Human	……	E	P	G	D	E	F	……
Rhesus	……	E	P	G	D	E	F	……
Mouse	……	E	P	G	D	E	F	……
Dog	……	E	P	G	D	E	F	……
Elephant	……	E	P	G	D	E	F	……
Opossum	……	E	P	G	D	E	F	……
Chicken	……	E	P	G	D	E	F	……
X-tropicalis	……	E	P	G	D	E	F	……
Zebrafish	……	Q	P	G	D	E	F	……

PRKAR1A amino acid sequence in adjacent region of p.289 glycine of multiple vertebrates; “……” means sequences omitted.

### 3.2. Prediction of Mutant PRKAR1A Protein Function

We investigated mutant PRKAR1A protein function prediction using the online protein prediction program Sift [15] and Polyphen [16]. Both of these predicted that the substitution of the amino acid glycine at codon 289 to glutamic acid would affect this protein’s function.

### 3.3. Ethics Statement 

We obtained approval for this study from the Medical Ethics Committee of the Peking Union Medical College Hospital. Written informed consent forms were signed by the patient’s parents and the healthy controls. All clinical investigations were conducted according to the principles of the Declaration of Helsinki.

## 4. Conclusions

We identified a missense *PRKAR1A* mutation in a Chinese acrodysostosis patient with concomitant multiple hormone resistance, which was the first *PRKAR1A* mutation identified in a Chinese acrodysostosis patient. Our results confirm that the p.G289E variation of *PRKAR1A* is responsible for acrodysostosis with concomitant multiple hormone resistance.

## Supplementary Files

Supplementary File 1
