# Supplementary material for: The First Mutation Identified in a Chinese Acrodysostosis Patient Confirms a p.G289E Variation of PRKAR1A Causes Acrodysostosis
Source: Int J Mol Sci. 2014 Jul 29;15(8):13267–74. doi: 10.3390/ijms150813267 (PMC4159793; doi:10.3390/ijms150813267)
Supplement: Supplementary File 1 [file ijms-15-13267-s001.pdf]

Supplementary Information

Table S1. Primers designed for PCR amplification and sequencing of *PRKAR1A* gene.

Primer Name	Primer Sequence (from 5' to 3')	Size (bp)/AT * (°C)
PRKAR1A-1F	GGCAGCGACAGGACTCTCCC	905/62.5
PRKAR1A-1R	GCACCAAAGCCAACGCCCAG	-
PRKAR1A-2F	TATTCCCTAGTCCCCACTTCC	572/53.5
PRKAR1A-2R	ACCCTTTATTAGCCAAGTACAGT	-
PRKAR1A-3F	TGCCCATTAATAAAGGATAGCAT	633/54.4
PRKAR1A-3R	GCAAGGAAATAACAAACCCCAA	-
PRKAR1A-4F	AGTTTTGCCTCAGAATAATGACC	845/53.5
PRKAR1A-4R	TCCCAATACAAAGTGTTCTGTC	-
PRKAR1A-5F	TTCCCCTGAAAGATTGTGT	481/55
PRKAR1A-5R	TTGAACTCCTGGGCTCAAGCA	-
PRKAR1A-6F	CATTTAACTCGTCAGAAATCACC	591/52
PRKAR1A-6R	ATGACCAGCACATATATACCAGA	-
PRKAR1A-7F	GTGATAGGCTCAGGTATAAACCG	464/51
PRKAR1A-7R	GAAGGCTTTTCCCAAGTCCAT	-
PRKAR1A-8F	AATCCTGCTCAGACTAGAGGT	381/53.5
PRKAR1A-8R	CTTTCCCTCTTAGAGCGTACAAC	-
PRKAR1A-9F	AGCACCTTAGCTTGATACGAA	594/53.5
PRKAR1A-9R	CACGAGGACGATTCATCAGT	-
PRKAR1A-10F	CCTGAATTTTATTTTCTAACTGC	1033/51
PRKAR1A-10R	AAAAGTATTTCAGAATTGGAG	-
PRKAR1A-11F	GTTGTTGTTTGCCAAGCTA	976/51
PRKAR1A-11R	AAAAATCTACCATGACCAA	-
PRKAR1A-12F	GGCACTTTTATTTTCATTGTGAT	988/51
PRKAR1A-12R	CCACCCGAGTAGACTTTCG	-

* AT: Anneal Temperature.
